# CSF pro-orexin and amyloid-β38 expression in Alzheimer's disease and frontotemporal dementia

**DOI:** 10.1016/j.neurobiolaging.2018.08.019

**Published:** 2018-12

**Authors:** Wendy E. Heywood, Jenny Hallqvist, Amanda J. Heslegrave, Henrik Zetterberg, Chiara Fenoglio, Elio Scarpini, Jonathan D. Rohrer, Daniela Galimberti, Kevin Mills

**Affiliations:** a Centre for Translational Omics, Genetics & Genomic Medicine, UCL Great Ormond Street Institute of Child Health, London, UK; b Department of Neurodegenerative Disease, UCL Institute of Neurology, Queen Square, London, UK; c UK Dementia Research Institute at UCL, London, UK; d Clinical Neurochemistry Laboratory, Sahlgrenska University Hospital, Mölndal, Sweden; e Department of Psychiatry and Neurochemistry, Institute of Neuroscience and Physiology, the Salhgrenska Academy at the University of Gothenburg, Sweden; f Neurodegenerative Disease Unit, University of Milan, Centro Dino Ferrari, Fondazione Cà Granda, IRCCS Ospedale Policlinico, Milan, Italy

**Keywords:** Alzheimer's disease, Frontotemporal dementia, Orexin, Progranulin, C9orf72, Amyloid β-38

## Abstract

There is an unmet need for markers that can stratify different forms and subtypes of dementia. Because of similarities in clinical presentation, it can be difficult to distinguish between Alzheimer's disease (AD) and frontotemporal dementia (FTD). Using a multiplex targeted proteomic LC-MS/MS platform, we aimed to identify cerebrospinal fluid proteins differentially expressed between patients with AD and FTD. Furthermore analysis of 2 confirmed FTD genetic subtypes carrying progranulin (*GRN*) and chromosome 9 open reading frame 72 (*C9orf72*) mutations was performed to give an insight into the differing pathologies of these forms of FTD. Patients with AD (n = 13) demonstrated a significant (*p* < 0.007) 1.24-fold increase in pro-orexin compared to FTD (n = 32). Amyloid beta-38 levels in patients with AD were unaltered but demonstrated a >2-fold reduction (*p* < 0.0001) in the FTD group compared to controls and a similar 1.83-fold reduction compared to the AD group (*p* < 0.001). Soluble TREM2 was elevated in both dementia groups but did not show any difference between AD and FTD. A further analysis comparing FTD subgroups revealed slightly lower levels of proteins apolipoprotein E, CD166, osteopontin, transthyretin, and cystatin C in the *GRN* group (n = 9) compared to the *C9orf72* group (n = 7). These proteins imply GRN FTD elicits an altered inflammatory response to *C9orf72* FTD.

## Graphical abstract

**Figure:**
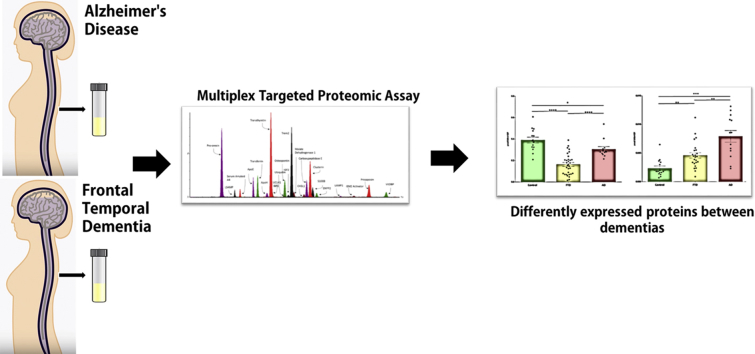

## 1 Introduction

It is estimated that over 850,000 people live with dementia in the UK alone and this figure is predicted to rise to over 2 million in the next 50 years. Research into new therapies and development of new drugs to stop or slow the progression of neurodegeneration is thus a major priority in health care. Development of treatment has been confounded in the past by difficulties in identifying the correct patients for new drug trials as it can be difficult clinically to distinguish between some forms as dementia such as Alzheimer's disease (AD) and frontotemporal dementia (FTD). Differential diagnosis between AD and FTD may be challenging as AD may manifest with behavioral disturbances (the “frontal variant” (Dubois et al., 2014)) whereas, on the other side, memory disturbances may manifest in FTD, particularly in carriers *of GRN* and chromosome 9 open reading frame (*C9orf72)* mutations (Galimberti et al., 2015, Pietroboni et al., 2011). Cerebrospinal fluid (CSF) biomarkers amyloid beta 1-42 (Aβ), total tau (tau), and Tau phosphorylated at position 181 (Ptau) have a good accuracy in predicting AD (Mulder et al., 2010). In clinical practice, this analysis helps to rule out AD, but apparently normal results cannot exclude FTD, as no specific biomarkers are available for this disease. Moreover, tau may be altered in FTD (as well as in many other neurodegenerative conditions), but surprisingly values are often normal in genetic forms, despite evidence of clinical deterioration (Carecchio et al., 2011). It is also possible that the CSF total- and phospho-tau (t-tau and p-tau, respectively) increase in AD is not a direct effect of tau pathology and neurodegeneration but rather reflects increased tau secretion from AD-affected neurons, as suggested in both animal studies (Maia et al., 2013) and a recent tau kinetics study in man (Sato et al., 2018). Further biomarkers are still needed as there is growing consensus that multiple biomarker panels may be the way forward in discriminating a molecular signature for individual dementias and their subtypes (Carecchio et al., 2011, Mulder et al., 2010). Targeted proteomics using multiple reaction monitoring liquid chromatography tandem mass spectrometry (MRM LC-MS/MS) is a useful technique in streamlining biomarker development. It does not require antibodies and can assay multiple proteins in a sample. Once a method is developed, it is capable of performing high-throughput analysis of multiple samples thereby enabling assessment of candidate markers. We have applied a previously described targeted proteomics platform of multiple markers of neurodegeneration in CSF from patients (Heslegrave et al., 2016a, Heywood et al., 2015, Paterson et al., 2016). We describe the use of this assay to analyze multiple biomarkers of neurodegeneration in the CSF from patients with AD, sporadic FTD, and 2 forms of genetic FTD (patients with mutations in *GRN* or *C9orf72*). We have looked for proteins that show a change between the 2 different dementias AD and FTD and also an analysis looking at proteins that show changes between sporadic and genetic subtypes of FTD. Looking at the differences between the different FTD groups will hopefully give an insight into the potential molecular pathology of these forms of FTD.

## 2 Methods

### 2.1 Ethics approval and consent to participate

Informed consent to participate in this study was given by all subjects or their caregivers. All samples were obtained from the Neurodegenerative Disease Unit of the Fondazione Cà Granda, IRCCS Ospedale Maggiore Policlinico, University of Milan (Milan, Italy).

### 2.2 CSF sample criteria, collection, and routine analysis

Sample data is given in Table 1. Patients were recruited consecutively at the Neurodegenerative Disease Center of the Ospedale Policlinico between 2013 and 2015. The clinical workup included medical history, physical and neurological examination, screening laboratory tests, neurocognitive evaluation, lumbar puncture for Aβ, tau, and Ptau evaluation, and imaging. Cognitive functions were assessed by the clinical dementia rating (CDR), the mini mental state examination (MMSE), the frontal assessment battery (FAB), the Wisconsin Card Sorting Test (WCST), and the Tower of London test. The presence of significant vascular brain damage was excluded (Hachinski Ischemic Score <4). The diagnosis of FTD was made according to consensus criteria (Neary et al., 1998) and subsequent revisions (McKhann et al., 2001, Rascovsky et al., 2011).

**Table 1:** Characteristics of AD patients, FTD patients, and controls

Subject details	Controls (n = 15)	AD patients (n = 13)	Sporadic FTD (n = 16)	C9orf72 FTD (n = 7)	GRN FTD (n = 9)
Gender (M/F)	5:10	5:8	10:6	6:1	3:6
Age, years (mean ± SD)	61.0 ± 9.9	72.1 ± 6.8	71.8 ± 7.3	66.0 ± 8.0	63.5 ± 8.5
APOE ε4 positive (%)	13.3%	69.2%	56.2%	14.2%	22%
CSF Biomarkers					
Aβ1-42 (pg/mL), median (IQR)	895 (794–1132)	446 (331.2–498.5)	656 (552.3–741.8)	839 (692–1141)	841 (687–1314)
T-tau (pg/mL), median (IQR)	91 ± (59-155)	481 (264–666)	688 (173–1114)	291 (239–367)	328 (268–440)
P-tau (pg/mL), median (IQR)	20.5 (17–28)	84 (56–105)	72 (28–107)	68 (38–84)	27(17–33)

Data expressed as mean ± SD or median (IQR) as appropriate.

Patients with AD (n = 13) all had abnormal CSF Aβ, t-tau, and p-tau levels, thus confirming the clinical diagnosis (G. McKhann et al., 1984) with an accuracy of about 90 % (Table 1), in accordance with more recent criteria (Dubois et al., 2010) (Dubois et al., 2007). Thirty-two patients with FTD were included: 7 *GRN* mutation carriers, 9 *C9orf72* expansion carriers, and 16 with sporadic disease.

Controls (n = 15) included patients undergone lumbar puncture on suspicion of neurological diseases that were disclosed with no evidence of neurological deficits and cognitive impairment. This cohort has previously been investigated for the effect of age on the proteins described in this study (Heywood et al., 2015). Only markers that did not show any significant change with age are presented in this study.

Clinical CSF was sampled according to a standard protocol (Blennow et al., 2010). CSF samples were obtained in polypropylene tubes by LP at the L4/L5 or L3/L4 interspace, centrifuged at 4 °C, and stored at  ≤ −30 °C until analysis. CSF cell counts, glucose, and proteins were determined. Routine analysis to exclude damage of the blood-brain barrier (BBB) included measurement of albumin by rate nephelometry and the intrathecal IgG production. The albumin quotient (CSF albumin/serum albumin) X 10^3^ and the IgG index (CSF albumin/serum albumin)/(CSF IgG/serum IgG) were calculated and samples without BBB damage were used in the study, which confirm that the proteins measured in this study are produced intrathecally and not leaking from the periphery (Sellebjerg and Christiansen, 1996).

### 2.3 Aβ, tau, and Ptau measurement

CSF tau concentration was determined using a sandwich enzyme-linked immunosorbent assay (ELISA) (INNOTEST hTAU-Ag, Fujirebio, Ghent, Belgium) specifically constructed to measure all tau isoforms irrespective of phosphorylation status. CSF Ptau was measured using a sandwich ELISA specifically detecting tau phosphorylated at amino acid 181 (INNOTEST PHOSPHO-TAU (181P), Fujirebio, Ghent, Belgium). Aβ levels were determined using a sandwich ELISA (INNOTEST ß- AMYLOID Fujirebio, Ghent, Belgium). Normal values of biomarkers were as follows: Aβ->550 pg/mL; tau  < 375 pg/mL; and Ptau  < 52 pg/mL (Mulder et al., 2010).

### 2.4 Targeted proteomics: MRM-based triple quadrupole mass spectral assay

A 54-protein MRM LC-MS/MS multiplex assay was originally designed to quantitate altered proteins that were identified from proteomic profiling of CSF from dementia patients as well as biomarker candidates suggested in the current literature at that time. Further detailed information regarding the selection and method development of the assay can be found in (Heywood et al., 2015). The initial analyses in this study were performed to identify proteins specific to the dementia type. Therefore, the FTD group comparison consisted of the *GRN*, *C9orf72* mutation carriers, and sporadic cases grouped together. Briefly, 20 nanograms of yeast enolase protein standard (Sigma, UK) and 10–50 pmols heavy labeled peptide standards (Thermo Scientific, UK) were added to 100 μL of CSF. CSF was freeze-dried and trypsin-digested as described previously (Heywood et al., 2012). A single 35 μL injection of each CSF digest was injected onto a Waters CORTECS UPLC C18 + Column, 90 Å, 1.6 μm, 3 mm  ×  100 mm column attached to a C18+ VanGuard precolumn. UPLC and MS tune conditions were performed as described previously (Manwaring et al., 2013). QC runs of pooled CSF digests were run in triplicate at the start of the run and then every 10 injections. A CV within ±15% for each QC was considered acceptable. Chromatograms were analyzed using Waters Targetlynx software. Peptides were standardized by either using a spiked heavy labeled peptide or to a yeast enolase peptide. Absolute levels of pmols/100 μL CSF were obtained from standard curves.

### 2.5 Statistical analysis

Analyses included data QC for peptide performance (coefficient of variance), QC of sample preparation, and LC-MS/MS performance (yeast enolase). The normality of the variables was evaluated using D'Agostino-Pearson's test. Non-normally distributed variables were transformed to Gaussian distribution by the operations listed in Supplementary Data 2. The differences in protein expression between the groups were evaluated using one-factor ANOVA in conjunction with Tukey's multiple comparison post-test or Mann-Whitney U nonparametric test. Group age ranges were checked for significant difference using a Kruskal-Wallis with Dunns post-test of which no significant difference was confirmed.

## 3 Results

### 3.1 Dementia grouped analysis

**Fig. 1:**
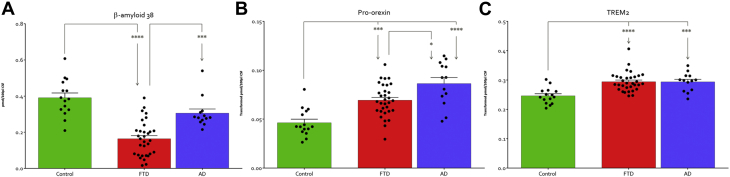
Proteins differently expressed between AD and FTD. (A) a-beta-38 (untransformed data) and (B) pro-orexin are significantly altered between FTD from AD. (C) sTREM2 is elevated in both AD and FTD but there is no difference between the dementias. * indicates *p* < 0.05, ***p < 0.001, ****p < 0.0001 as determined by nonparametric Mann-Witney U analysis. Key: AD, Alzheimer's disease; FTD, frontotemporal dementia; sTREM2, soluble TREM2.

Only 2 proteins showed significant changes when samples were grouped according to dementia type (Fig. 1). Aβ-38 demonstrated a slight but not significant reduction in the mean of the AD group. However, the FTD group demonstrated a much greater and significant 2.6-fold reduction compared with controls (*p* < 0.0001) and a significant 1.83-fold reduction compared to the AD group (*p* < 0.001). Pro-orexin levels were statistically and significantly increased in both dementia groups with a 1.5-fold increase in FTD compared to controls (*p* < 0.001) and a 1.87-fold increase in the AD group (*p* < 0.0001). The AD group also demonstrated a significant 1.24-fold higher level of pro-orexin when compared to the FTD group (*p* < 0.01). Soluble TREM2 (sTREM2) was included in the analysis and has previously been demonstrated to be elevated in AD using our assay (Heslegrave et al., 2016b) and been shown to be elevated previously in FTD CSF by ELISA (Piccio et al., 2016). CSF levels of sTREM2 were significantly elevated in both the AD and FTD groups relative to the control group (*p* < 0.0006). sTREM2 levels although elevated in both the AD and FTD groups were not different between both the neurodegenerative conditions. Correlation analysis with levels of routinely measured known disease CSF markers amyloid Aβ, tau, and Ptau did not demonstrate any relationship with pro-orexin or sTREM2 levels in CSF of either dementia. There was significant correlation between levels of Aβ-38 with Ptau in AD (r^2^ = 0.37 *p* < 0.028) but not FTD. However, Aβ-38 did show a correlation with Aβ-42 in the FTD group (r^2^ = 0.38, *p* < 0.001) (Supplementary Data 1).

### 3.2 FTD subgroup analysis

**Fig. 2:**
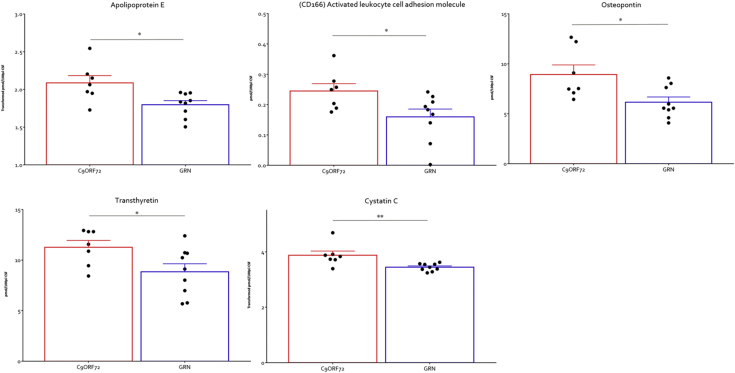
Proteins altered between the C9orf72 and GRN genetic forms of FTD. * indicates *p* < 0.05, ** *p* < 0.01 as determined by nonparametric Mann-Witney U analysis. Key: FTD, frontotemporal dementia.

The FTD group was subdivided into 2 groups consisting of those patients carrying *GRN* mutations and those with *C9orf72* expansions. Five proteins showed small but significant changes (*p* < 0.04–0.01) between the *GRN* and *C9orf72* groups. The proteins in the *GRN* group which consistently showed a lower level relative to the C9orf72 group were apolipoprotein E, CD166, transthyretin, osteopontin, and cystatin C (Fig. 2).

## 4 Discussion

In previous work by our laboratory, we demonstrated that our method revealed significantly elevated proteins in the CSF of patients with AD relative to controls. Therefore, the aim of this study was to determine if our test was capable of detecting proteins altered between AD and FTD and if it was possible to see changes between FTD carrying different causal mutations. We found that only 2 proteins (pro-orexin and Aβ-38) differed in expression between the AD and FTD groups, whereas 5 proteins were altered between carriers of different mutations in FTD.

Analysis of pro-orexin levels revealed that this protein was slightly significantly elevated in AD compared to patients with FTD. Pro-orexin has been implicated previously in the pathogenesis of AD (Liguori et al., 2014, Malkki, 2014) and in particular the hypothalamic dysfunction related to sleep impairment in AD (Liguori et al., 2017). Sleep disturbance can also be observed in FTD albeit not as commonly (McCarter et al., 2016). Consistent with this, we observed raised CSF pro-orexin in FTD compared with controls but to a lesser extent than AD (Coban et al., 2013). It would be useful in future studies to correlate the presence of sleep symptoms with pro-orexin levels to see whether abnormalities in this protein can predict the presence of sleep disturbance.

The finding of reduced Aβ-38 levels specifically in the FTD group confirms previous observations by other groups (Bibl et al., 2011, Gabelle et al., 2011). However, the reason for this remains unclear. Although AD pathology can occasionally be seen as an additional finding at postmortem in patients with FTD, amyloid has not been implicated more generally in the pathophysiology of the FTD syndromes and Aβ-38 is not a plaque-associated form of Aβ. The levels of Aβ-42 and Aβ-40 overall are not altered to the degree as that seen for Aβ-38 in the FTD group. This indicates the effect on Aβ-38 is downstream of this mechanism and likely due to increased proteolysis. Aβ-38 can be cleaved by other enzymes such as neprilysin, insulin-degrading enzyme, endothelin-converting enzyme, angiotensin-converting enzyme, and the plasmin/uPA/tPA (Wang et al., 2006), and potentially, Aβ-38 is a better substrate for these proteases than the other Aβ peptides. Interestingly, some other substrates of these proteases particularly neprilysin are feeding hormones. Alterations in feeding behavior are a known clinical feature in some forms of FTD (Woollacott and Rohrer, 2016). It is possible that dysregulation of such a protease could be an initiating factor for some of the downstream pathological features in FTD. Neprilysin has been studied in the context of AD more for its potential as a plaque treatment (Meilandt et al., 2009) but so far neprilysin has not been investigated in FTD. Another theoretical explanation for the more pronounced decrease of Aβ-38 in FTD could be γ-secretase modulation; decreased processivity of APP by γ-secretase could result in lower concentrations of shorter Aβ peptides, such as Aβ38 (Chavez-Gutierrez et al., 2012), but this has to the best of our knowledge not been studied in FTD. We did attempt to look at proteins that could be altered just in sporadic FTD but no specific changes were observed apart from the previous pro-orexin and amyloid beta 38. Because of the heterogeneity of FTD pathology, this is unsurprising. Until further understanding of sporadic FTD at a molecular level is understood, it is hard to identify proteins that indicate specific subpathologies.

When subgrouping the FTD patients by genetic status, 5 proteins measured in the multiplex assay demonstrated a small but significant difference between the 2 genetic FTD groups, with lower levels in the *GRN* group compared with *C9orf72* group. Reduced expression of cystatin C has been described in response to systemic inflammation in dendritic cells (Xu et al., 2011). This is consistent with previous work suggesting differential involvement of inflammatory pathways in *GRN*-related FTD compared with other subtypes of FTD (Martens et al., 2012). However, the other proteins (osteopontin, CD166, transthyretin, apolipoprotein E) have also been postulated to be involved in the inflammatory response (Bowen and Aruffo, 1999, Rebeck, 2017, Shin, 2012) but this does not corroborate with *GRN* neuropathology which is found to be more profound than that observed in *C9orf72* pathology. One reasoning for this observation could be that these proteins maybe reduced in *GRN* CSF due to being sequestered at sites of inflammation in the brain. Further work should aim to replicate these measures in a larger FTD data set and understand further their exact role in FTD.

This study shows that a targeted MRM platform measuring multiple proteins are able to not only help to find proteins with altered expression between AD from FTD but can also identify differential involvement of proteins in FTD subtypes, consistent with the different pathophysiological mechanisms underlying these different forms of dementia. Limitations of this study include (1) the modest number of samples; and (2) the lack of pathological demonstration of the pathology, particularly in sporadic FTD, for which no biomarkers exist. As with discovery of all potential biomarkers, independent validation on separate and much larger cohorts will be required for any future clinical utilization. We believe this initial analysis and platform are a valuable proviso toward achieving this further validation in larger cohorts of clinically well-defined samples. Another strength of this platform is that other possible candidate biomarkers suggested from other studies and even possibly neprilysin from our own findings could be added to the panel.

## Disclosure statement

The authors have no actual or potential conflicts of interest.

## Appendix A Supplementary data

Data Profile

Supplementary data

CSF Multiplex Assay Data
