# Supplementary material for: CSF pro-orexin and amyloid-β38 expression in Alzheimer's disease and frontotemporal dementia
Source: Neurobiol Aging. 2018 Dec;72:171–6. doi: 10.1016/j.neurobiolaging.2018.08.019 (PMC6221294; doi:10.1016/j.neurobiolaging.2018.08.019)
Supplement: Supplementary data [file mmc2.docx]

CSF pro-orexin and amyloid β-38 distinguish Alzheimer’s disease from frontotemporal dementia

Wendy E. Heywood ^1^, Amanda J. Heslegrave ^2,3^, Henrik Zetterberg ^2,3,4,5^, Chiara Fenoglio^3^, Elio Scarpini^3^, Jonathan D. Rohrer^2^, Daniela Galimberti^3*^, Kevin Mills^1*§^

^1^Centre for Translational Omics, Genetics & Genomic Medicine, UCL Great Ormond Street Institute of Child Health, 30 Guilford St London, UK

^2^ Department of Molecular Neuroscience, UCL Institute of Neurology, Queen Square, London WC1N 3BG, UK

^3^ UK Dementia Research Institute at UCL, London WC1N 3BG, UK

^4^ Clinical Neurochemistry Laboratory, Sahlgrenska University Hospital, Mölndal, S-431 80 Mölndal, Sweden

^5^ Department of Psychiatry and Neurochemistry, Institute of Neuroscience and Physiology, the Salhgrenska Academy at the University of Gothenburg, S-431 80 Mölndal, Sweden

^3^ Neurology Unit, Department of Pathophysiology and Transplantation, University of Milan, Fondazione Cà Granda, IRCCS Ospedale Policlinico, Milan, Italy

*Authors have contributed equally to this work,

§ Corresponding author: Dr Kevin Mills, Centre for Translational Omics, UCL Great Ormond Street Institute of Child Health, London, UK. kevin.mills@ucl.ac.uk

**Supplementary data 1. Correlation analysis of identified proteins with ELISA values of existing markers of dementia.** orexin does not show any correlation with i) Aβ-42 ii) phosphorylated tau or iii) total tau levels. Aβ-38 shows correlation in the FTD group with iv) Aβ-42 (r^2^= 0.38 p<0.001) and v) in the AD group with phosphorylated tau (r^2^= 0.36 p<0.028). no correlation is observed for Aβ-38 against total tau levels.

Supplementary data 1.
